# The Expression Levels and Significance of GSH, MDA, SOD, and 8-OHdG in Osteochondral Defects of Rabbit Knee Joints

**DOI:** 10.1155/2022/6916179

**Published:** 2022-01-19

**Authors:** Zhengjun Xiao, Xuefeng Yu, Shuai Zhang, A. Liang

**Affiliations:** ^1^Orthopedics Department, Affiliated Central Hospital of Shenyang Medical College, 110020, China; ^2^Orthopedics Department, Xinhua Hospital Affiliated to Dalian University, 116000, China; ^3^Orthopedics Department, The First Hospital of China Medical University, 110001, China

## Abstract

**Objective:**

To observe the dynamic changes in oxidative stress and the expression levels of antioxidant and oxidative parameters in the blood and synovial fluid in the osteochondral defects of the rabbit knee joints and to explore the significance.

**Methods:**

Thirty New Zealand white rabbits were randomly selected and divided into a blank control group (*n* = 10), a model control group (*n* = 10), and an osteochondral defect group (*n* = 10). The osteochondral defect model of rabbit knee joint was constructed by medial parapatellar arthrotomy. The expression levels of glutathione (GSH), malondialdehyde (MDA), superoxide dismutase (SOD), and 8-hydroxydeoxyguanosine (8-OHdG) in peripheral venous blood and knee synovial fluid of the three groups were measured at the end of the 4th, 8th, and 12th weeks after treatment.

**Results:**

The expression levels of GSH and SOD in the blood and synovial fluid in the osteochondral defect group at the end of the 8th and 12th weeks were observably lower than those of the other two groups (*P* < 0.05); higher expression levels of MDA and 8-OHdG in the blood and synovial fluid of the osteochondral defect group compared with those of the other two groups were obtained (*P* < 0.05). At the end of the 4th, 8th, and 12th weeks, the expression levels of MDA and 8-OHdG in the blood and synovial fluid of the osteochondral defect group presented an upward trend (*P* < 0.05).

**Conclusion:**

The osteochondral defects initiate the oxidative stress in the body, which is presented as the decrease of GSH and SOD expression, and the upregulation of MDA and 8-OHdG expression.

## 1. Introduction

Osteoarthritis (OA) is the most common joint degenerative disease in the world, to which the middle-aged and elderly population are more susceptible [1]. More than 50% of the elderly in China suffer from OA, and its incidence has been presenting a gradual rising trend with the aging of population [2]. With main symptoms of knee joint pain and limited mobility, OA is the major cause of disability in the elderly, which, as a result, seriously drags down patients' quality of life [3] and also imposes a heavy economic burden on them [4]. Articular cartilage degradation is one of the manifestations of OA progression [5], and inflammation and metabolic factors are considered an essential pathological basis of OA. Oxidative stress results from an imbalance between the oxidative and antioxidant systems of cells and tissues and is the result of excessive production of oxidative free radicals and related reactive oxygen species (ROS). A large number of studies have confirmed a close relationship of the oxidative stress triggered by the slow production of endogenous ROS to the osteochondral defects [6, 7]. To be specific, studies have shown that excessively generated free radicals give rise to osteochondrocyte apoptosis and degrade the extracellular matrix, which eventually results in osteochondral defects [8, 9]. GSH, MDA, SOD, and 8-OHdG are the main biomarkers of oxidative stress. The current research interest lies more on the relationship between oxidative stress and the process of osteochondral defects at a certain time point. However, researches on the dynamic observation of the expression levels and significance of antioxidant and oxidative biomarkers in blood and synovial fluid are little, if anything, to be found. Therefore, this study is to preliminarily explore the relationship between osteochondral defects and oxidative stress in rabbit knee joints and evaluate the effect of osteochondral defect repair on the expression of antioxidant and oxidative biomarkers in the blood and synovial fluid, with a view to providing a reliable theoretical basis for clinical treatment in the future.

## 2. Materials and Methods

### 2.1. Material

#### 2.1.1. Experimental Animals

Thirty New Zealand rabbits, specific pathogen-free (SPF), 6 months old, 15 males and 15 females, weighing 3.0-3.5 kg, were purchased from Chongqing Weston Biomedical Technology Co., Ltd. During the experiment, standard laboratory diet and drinking water were given. After 1 week of adaptive feeding, the animals were randomly divided into a blank control group (*n* = 10), a model control group (*n* = 10), and an osteochondral defect group (*n* = 10). Laboratory animal disposal complied with animal ethics requirements.

#### 2.1.2. Main Reagents

The following materials and equipment were used in the study: SOD kit: Nanjing Jiancheng Biotechnology Co., Ltd., article number A001-3-1; MDA kit: Nanjing Jiancheng Biotechnology Co., Ltd., article number A003-1; reduced GSH kit: Jiangxi Aiboin Biotechnology Co., Ltd., item number IB-E22122; 8-OHdG kit: Shanghai Lianmai Bioengineering Co., Ltd., item number LM-8-OHdG-Ge; ultraviolet-visible spectrophotometer: Shanghai Unico Instrument Co., Ltd., model UV-2100PC; and microplate reader: Thermo Fisher Company, USA, model Multiskan MS.

### 2.2. Method

#### 2.2.1. Model Construction

The animal model of rabbit knee joint osteochondral defects was prepared using the modeling method of Elwan et al. [10]. Before surgery, venous access was established in the ear veins of each animal. After obtaining a satisfactory intravenous anesthesia (corneal reflex disappeared and the breathing became stable) with 3% thiopental sodium (20~40 mg/kg), thiopental sodium 3~5 mg/kg was added intravenously every 20~30 minutes to maintain the anesthesia effect. In the surgery process, the use of additional doses was an option according to the rabbit's performance. After all rabbits' left knee joints in the model control group and the osteochondral defect group were prepared for the surgery, a skin incision was performed on the medial side of the knee joint, and medial parapatellar arthrotomy was performed to deviate the patella laterally and expose the articular surface of the femoral condyle. In the osteochondral defect group, a drill was used to obtain a cylindrical full-thickness osteochondral defect with a diameter of 1.5 mm and a depth of 2 mm on the anterior side of the articular surface of the femoral condyle, while no osteochondral defect treatment was given to the model control group. After the osteochondral defects in the osteochondral defect group was formed, the joints of the model control group and the osteochondral defect group were washed with normal saline, and the joint capsules were routinely closed. After the operation, the rabbit's knee joint could move freely without fixation. In addition, each animal was given regular intraperitoneal injections of buprenorphine for postoperative analgesia, and no treatment was given to the blank control group. After 3 months of follow-up, the animals were euthanized by injecting air into the ear vein.

#### 2.2.2. Blood Collection

The partial coat on the rabbit ear veins was removed, the skin was disinfected with 75% alcohol, and 2 mL of blood from the rabbit ear veins was collected by puncturing the end of the ear vein with a needle. The collection time was at the end of the 4th week, the 8th week, and the 12th week after the operation. The blood was placed still at room temperature for 1 hour and centrifuged at 3000 r/min for 10 minutes at 4°C, and the serum was collected and stored in a refrigerator at -80°C. The serum was assayed for oxidation and antioxidant biomarkers, including GSH, SOD, MDA, and 8-OHdG.

#### 2.2.3. Synovial Fluid Collection

The animal was placed on a fixed table and the fur around the puncture point of the left knee joint was cleaned. The puncture site was disinfected, the rabbit's knee joint was straightened, and an oblique puncture from the lateral side of the patella was performed. The puncture was stopped after the needle reached the joint cavity where no pressure on the needle was received and no blood could be drawn. 1 mL of normal saline was injected into the joint cavity by using a 5 mL syringe, the knee joint was exercised manually several times, and then, about 0.5-1 mL of synovial fluid was drawn. The collection time was at the end of the 4th week, the 8th week, and the 12th week after the operation. The synovial fluid was centrifuged at 3000 r/min for 5 minutes, the supernatant was collected and stored at -80°C, and the levels of GSH, SOD, MDA, and 8-OHdG were detected.

### 2.3. Statistical Analysis

The SPSS 21.0 (SPSS Inc., for Windows) software was used to analyze the data obtained from the study. The measurement data conforming to the normal distribution were represented as ($\bar{\text{x}}\pm \text{SD}$), the three groups were compared by one-way analysis of variance, and the SNK-*q* test was used for pairwise comparison.

## 3. Results

### 3.1. The Expression Levels of Oxidative and Antioxidant Biomarkers in the Three Groups of Synovial Fluid and Blood before Surgery

No significant difference in the levels of GSH, MDA, SOD, and 8-OhdG in the synovial fluid and blood of the three groups before operation was found (*P* > 0.05). See Tables 1 and 2.

### 3.2. Expression Levels of Oxidative and Antioxidant Biomarkers of the Three Groups at the End of the Fourth Week

Compared with the blank control group, the model control group and the osteochondral defect group yielded a downward trend of the GSH and SOD in the blood and synovial fluid and an upward trend of the MDA and 8-OHdG at the end of the fourth week; however, the differences were not considered statistically significant (*P* > 0.05), as shown in Tables 3 and 4.

### 3.3. Expression Levels of Oxidative and Antioxidant Biomarkers of the Three Groups at the End of the Eighth Week

In contrast to the blank control group, apparently lower levels of GSH, MDA, SOD, and 8-OHdG in the synovial fluid and blood of the other two groups at the 8th week were observed (*P* < 0.05). See Tables 5 and 6.

### 3.4. Expression Levels of Oxidative and Antioxidant Biomarkers of the Three Groups at the End of the Twelfth Week

Tables 7 and 8 demonstrate that compared to the blank control group, the other two groups garnered noticeably lower levels of GSH, MDA, SOD, and 8-OHdG in the synovial fluid and blood at the twelfth week (*P* < 0.05).

### 3.5. Changes in the Expression Levels of Oxidative and Antioxidant Biomarkers in Osteochondral Defects

**Figure 1:**
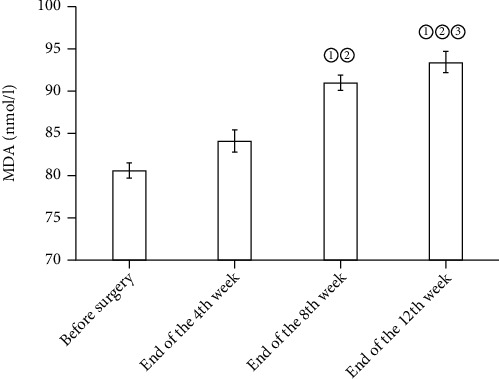
Expression levels of MDA in synovial fluid before operation at the end of the 4th, 8th, and 12th weeks. Note: compared with before surgery, ^①^*P* < 0.05; compared with the end of the 4th week, ^②^*P* < 0.05; compared with the end of the 8th week, ^③^*P* < 0.05.

**Figure 2:**
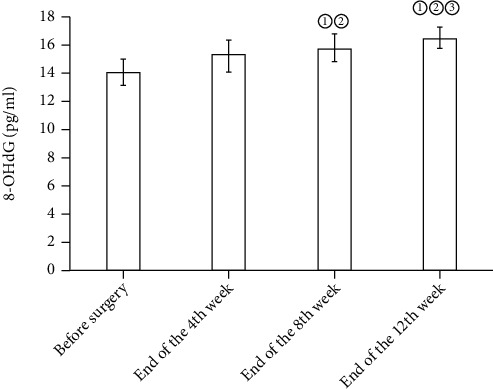
Expression levels of 8-OHdG in synovial fluid before operation at the end of the 4th, 8th, and 12th weeks. Note: compared with before surgery, ^①^*P* < 0.05; compared with the end of the 4th week, ^②^*P* < 0.05; compared with the end of the 8th week, ^③^*P* < 0.05.

**Figure 3:**
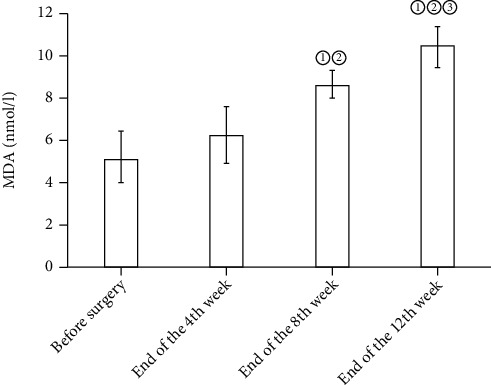
Expression levels of MDA in blood before operation at the end of the 4th, 8th, and 12th weeks. Note: compared with before surgery, ^①^*P* < 0.05; compared with the end of the 4th week, ^②^*P* < 0.05; compared with the end of the 8th week, ^③^*P* < 0.05.

**Figure 4:**
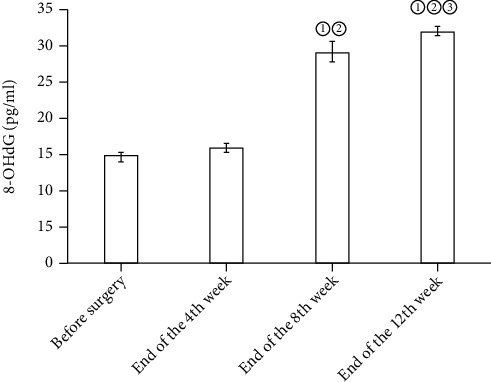
Expression levels of 8-OHdG in blood before operation at the end of the 4th, 8th, and 12th weeks. Note: compared with before surgery, ^①^*P* < 0.05; compared with the end of the 4th week, ^②^*P* < 0.05; compared with the end of the 8th week, ^③^*P* < 0.05.

In the osteochondral defect group, GSH and SOD in the blood and synovial fluid before surgery and at the end of the fourth, eighth, and twelfth weeks rose gradually, yet no statistical differences were detected (*P* > 0.05). Compared with before surgery, the expression levels of MDA and 8-OHdG in the synovial fluid and blood saw a surge at the end of the 8th and 12th weeks (*P* < 0.05). See Figures 1, 2, 3, and 4.

## 4. Discussion

With the aging of population, the incidence of OA has been presenting a rising trend, with osteochondral degradation being the most quintessential pathological change. Oxidative stress is involved in the pathogenesis of hypertension, atherosclerosis, diabetes, osteoporosis, and cancer. It is also known to cause and accelerate the aging process. Although exercise of the knee joint has numerous benefits for human health, it is also often claimed to induce oxidative stress since exercise increases ROS generation. Several studies have demonstrated that exercise increases the plasma 8-OHdG level. As mentioned above, oxidative stress plays a crucial role in the development of osteochondral degradation [6, 7], the degree of which in the body is described by dynamic changes of major oxidative stress biomarkers. Antioxidant capacity is usually evaluated by determining the activity of antioxidant enzymes [11]. SOD, an essential antioxidant enzyme in the body, and GSH, an important enzyme that catalyzes the decomposition of hydrogen peroxide, are two paramount substances in cell defense [12, 13]; 8-OHdG, the product of DNA oxidative damage induced by free radicals, is widely applied as a biomarker of oxidative stress and damage [14].

This study explored the dynamic changes of oxidative stress during the repair of osteochondral defects in the classic animal model of knee osteochondral defects in New Zealand white rabbits. We found that at the end of the fourth week, there was no significant difference in the expression levels of GSH, MDA, SOD, and 8-OHdG among the three groups, which suggested a similar condition without significant difference in terms of degree of oxidative stress among the three groups. However, at the end of the 8th and 12th weeks, compared with the other two groups, the expression levels of MDA and 8-OHdG in the osteochondral defect group witnessed an elevation, while the expression levels of GSH and SOD plummeted, which indicated that osteochondral defects gave rise to an upregulation of oxidative stress in the body. Ostalowska et al. [15] found an aberrant antioxidant status in the synovial fluid of patients with gonarthritis. The course of OA interferes with the articular cartilage and oxidative stress, resulting in decreased levels of GSH and SOD [16, 17]. It has also pointed out that in the process of inflammation, the activation of oxidative stress is invariably accompanied by the formation of free radicals, which plays a major role in disease progression and subsequent destruction of articular cartilage [18]. It is consistent with the results at the end of the eighth and twelfth weeks in this study. Reasons for the results at the end of the fourth week where no difference among the three groups was found can be that on the one hand, the test efficiency suffered a setback in consequence of the small size of samples; on the other hand, the differences of oxidant stress among the three groups did not begin to present any statistical significance at that time point. The production of ROS is induced by the stimulation by inflammatory cytokines on chondrocytes [19, 20]. These studies have thus confirmed the significance of reducing the level of oxidative stress in chondrocytes [7]. In addition, we found that over time, the repair of osteochondral defects led to an upward trend of oxidative biomarkers in the blood and synovial fluid, while no obvious abnormality was seen in the antioxidant biomarkers, which demonstrated that the degree of oxidative stress persists and gradually aggravates over time during the repair process. However, the sex effect was not studied in the present study, which is expected to be explored in the future.

In summary, the repair process of osteochondral defects slowly initiates the body's oxidative stress, which upregulates the expression levels of oxidation markers but inhibits the expression levels of antioxidant markers in the body. It implies that the intervention of the degree of oxidative stress in the repair of osteochondral defects may be of great significance to the diagnosis, treatment, and prognosis of OA. Recent studies have shown that the activation of adenylate-activated protein kinase impedes the viability of osteoblasts under oxidative stress conditions, which in turn hinders the balance of bone metabolism [21]. Therefore, on the basis of this research, we plan to further explore the role of related molecular mechanisms in future research. Finally, a number of importance limitations need to be considered: (1) The setting of time gradients needs further exploration. In future studies, multiple sets of time gradients will be set to dynamically observe the expression levels and significance of oxidative stress biomarkers in the process of osteochondral defect repair. (2) The molecular signaling pathway of oxidative stress in the process of osteochondral defect repair has not been studied.

## Figures and Tables

**Table 1:** The expression levels of GSH, MDA, SOD, and 8-OHdG in the synovial fluid of the three groups before surgery ($\bar{\text{x}}\pm \text{s}$).

Groups	GSH (nmol/mL)	SOD (U/mL)	MDA (nmol/L)	8-OHdG (pg/mL)
Blank control group	92.28 ± 0.63	42.58 ± 1.02	84.02 ± 1.83	14.37 ± 0.88
Model control group	91.85 ± 0.51	36.36 ± 1.28	83.74 ± 1.59	14.00 ± 0.82
Osteochondral defect group	92.93 ± 0.91	39.94 ± 1.42	80.59 ± 0.85	14.25 ± 1.03
*F*-value	23.540	29.031	26.269	30.013
*P* value	0.323	0.173	0.269	0.162

**Table 2:** The expression levels of GSH, MDA, SOD, and 8-OHdG in the blood of the three groups before surgery ($\bar{\text{x}}\pm \text{s}$).

Groups	GSH (nmol/mL)	SOD (U/mL)	MDA (nmol/L)	8-OHdG (pg/mL)
Blank control group	43.27 ± 0.84	20.43 ± 1.53	4.57 ± 1.42	15.49 ± 1.20
Model control group	41.68 ± 1.36	18.34 ± 1.72	6.24 ± 1.52	14.31 ± 1.32
Osteochondral defect group	42.61 ± 1.03	19.19 ± 1.39	5.29 ± 1.26	14.82 ± 0.62
*F*-value	29.418	28.694	26.677	27.330
*P* value	0.142	0.150	0.182	0.167

**Table 3:** Expression levels of GSH, MDA, SOD, and 8-OHdG in the synovial fluid of the three groups at the end of the fourth week ($\bar{\text{x}}\pm \text{s}$).

Groups	GSH (nmol/mL)	SOD (U/mL)	MDA (nmol/L)	8-OHdG (pg/mL)
Blank control group	92.13 ± 1.16	42.41 ± 1.20	85.53 ± 1.32	14.95 ± 0.96
Model control group	92.48 ± 1.30	36.74 ± 0.89	83.08 ± 1.27	14.70 ± 1.10
Osteochondral defect group	90.32 ± 1.24	40.98 ± 1.23	84.04 ± 1.33	15.38 ± 1.16
*F*-value	10.490	11.537	12.624	8.226
*P* value	0.073	0.069	0.082	0.162

**Table 4:** Expression levels of GSH, MDA, SOD, and 8-OHdG in the blood of the three groups at the end of the fourth week ($\bar{\text{x}}\pm \text{s}$).

Groups	GSH (nmol/mL)	SOD (U/mL)	MDA (nmol/mL)	8-OHdG (pg/mL)
Blank control group	43.08 ± 1.30	22.50 ± 1.12	4.82 ± 0.93	15.42 ± 0.68
Model control group	40.72 ± 1.46	17.16 ± 1.30	6.21 ± 0.82	14.22 ± 0.72
Osteochondral defect group	38.82 ± 0.73	18.09 ± 1.46	6.37 ± 1.40	16.04 ± 0.80
*F*-value	29.362	11.746	28.658	7.549
*P* value	0.051	0.068	0.059	0.092

**Table 5:** Expression levels of GSH, MDA, SOD, and 8-OHdG in the synovial fluid of the three groups at the end of the eighth week ($\bar{\text{x}}\pm \text{s}$).

Groups	GSH (nmol/mL)	SOD (U/mL)	MDA (nmol/L)	8-OHdG (pg/mL)
Blank control group	91.46 ± 1.29	43.07 ± 1.20	82.49 ± 1.30	14.82 ± 1.26
Model control group	91.89 ± 0.85	42.48 ± 1.16	81.10 ± 0.87	14.99 ± 1.25
Osteochondral defect group	86.63 ± 1.16^①②^	38.09 ± 0.80^①②^	90.92 ± 0.94^①②^	16.01 ± 1.00^①②^
*F*-value	30.537	26.468	25.836	27.664
*P* value	0.013	0.029	0.031	0.026

Note: compared with the blank control group, ^①^*P* < 0.05; compared with the model control group, ^②^*P* < 0.05.

**Table 6:** Expression levels of GSH, MDA, SOD, and 8-OHdG in the blood of the three groups at the end of the eighth week ($\bar{\text{x}}\pm \text{s}$).

Groups	GSH (nmol/mL)	SOD (U/mL)	MDA (nmol/mL)	8-OHdG (pg/mL)
Blank control group	43.22 ± 1.16	21.27 ± 1.31	4.50 ± 1.04	15.75 ± 1.16
Model control group	41.89 ± 0.93	17.83 ± 1.04	6.20 ± 0.80	13.41 ± 1.40
Osteochondral defect group	37.2 ± 0.88^①②^	11.62 ± 1.42^①②^	18.75 ± 0.70^①②^	29.6 ± 1.34^①②^
*F*-value	27.835	15.376	24.209	29.647
*P* value	0.028	0.051	0.043	0.017

Note: compared with the blank control group, ^①^*P* < 0.05; compared with the model control group, ^②^*P* < 0.05.

**Table 7:** Expression levels of GSH, MDA, SOD, and 8-OHdG in the synovial fluid of the three groups at the end of the twelfth week ($\bar{\text{x}}\pm \text{s}$).

Groups	GSH (nmol/mL)	SOD (U/mL)	MDA (nmol/L)	8-OHdG (pg/mL)
Blank control group	93.45 ± 1.07	42.58 ± 1.30	84.04 ± 1.09	14.02 ± 1.26
Model control group	91.85 ± 1.36	41.69 ± 0.75	83.84 ± 1.15	13.29 ± 0.83
Osteochondral defect group	83.16 ± 0.94^①②^	36.83 ± 1.20^①②^	93.3 ± 1.25^①②^	16.76 ± 0.82^①②^
*F*-value	25.398	32.007	37.068	26.640
*P* value	0.047	0.002	<0.001	0.003

Note: compared with the blank control group, ^①^*P* < 0.05; compared with the model control group, ^②^*P* < 0.05.

**Table 8:** Expression levels of GSH, MDA, SOD, and 8-OHdG in the blood of the three groups at the end of the twelfth week ($\bar{\text{x}}\pm \text{s}$).

Group	GSH (nmol/mL)	SOD (U/mL)	MDA (nmol/mL)	8-OHdG (pg/mL)
Blank control group	45.91 ± 0.79	21.04 ± 1.25	4.54 ± 0.93	15.57 ± 1.36
Model control group	42.05 ± 0.94	18.9 ± 1.39	6.28 ± 0.87	14.17 ± 1.52
Osteochondral defect group	32.68 ± 0.90^①②^	10.52 ± 1.55^①②^	10.59 ± 1.00^①②^	32.37 ± 0.59^①②^
*F*-value	28.185	29.530	39.502	40.284
*P* value	0.032	0.011	0.001	<0.001

Note: compared with the blank control group, ^①^*P* < 0.05; compared with the model control group, ^②^*P* < 0.05.

## Data Availability

The data used to support the findings of this study are available from the corresponding author upon request.
